# MicroRNAs in the Neural Retina

**DOI:** 10.1155/2014/165897

**Published:** 2014-03-05

**Authors:** Kalina Andreeva, Nigel G. F. Cooper

**Affiliations:** Department of Anatomical Sciences and Neurobiology, School of Medicine, University of Louisville, 500 S. Preston Street, Louisville, KY 40292, USA

## Abstract

The health and function of the visual system rely on a collaborative interaction between diverse classes of molecular regulators. One of these classes consists of transcription factors, which are known to bind to DNA and control the transcription activities of their target genes. For a long time, it was thought that the transcription factors were the only regulators of gene expression. More recently, however, a novel class of regulators emerged. This class consists of a large number of small noncoding endogenous RNAs, namely, miRNAs. The miRNAs compose an essential component of posttranscriptional gene regulation, since they ultimately control the fate of gene transcripts. The retina, as a part of the central nervous system, is a well-established model for unraveling the molecular mechanisms underlying neuronal and glial functions. Numerous recent efforts have been made towards identification of miRNAs and their inferred roles in the visual pathway. In this review, we summarize the current state of our knowledge regarding the expression and function of miRNA in the neural retina and we discuss their potential uses as biomarkers for some retinal disorders.

## 1. Introduction

The neural retina is an established model for unraveling the molecular mechanisms behind neuronal crosstalk. The initiation of the visual processing arises in the retina and, in accordance with its complex physiology, a broad number of coding genes have been detected and associated with different developmental stages, cellular components, and disorders of the retina. Now, we are beginning to understand the contribution of the noncoding regions of the genome to vision and its disturbances. In fact, only 1.5% of the human genome encodes proteins, so that the majority of DNA is noncoding. A portion of the noncoding DNA gives rise to various noncoding RNA transcripts, and their role is just starting to be unveiled. Among the different noncoding transcripts, microRNAs (miRNAs; miRs) are perhaps the best characterized class and possess a deep evolutionary history [1]. It is thought that approximately 1% of the human genome consists of miRNA genes [2]. MiRNAs are endogenous, noncoding, regulatory RNA molecules that are 19–23 nucleotides long and typically act through downregulation of their target coding transcripts in a sequence specific manner. MicroRNAs originate from long endogenous transcripts which fold back into hairpin-like structures. The transcripts undergo several processing steps and engage large protein complexes that yield mature miRNAs. In this mature stage, miRNAs are loaded into a RNA-induced silencing complex (RISC) and bind predominantly to the 3'UTR of complementary target mRNAs using a 6–8 nucleotides stretch known as the seed sequence [3]. Depending on sequence complementarity and most likely other as yet unknown mechanisms, this binding either obstructs the protein translational machinery and/or induces mRNA decay [4], indicating, therefore, the fate of the gene transcripts [5].

MiRNAs constitute an essential component of gene regulation and control well over half of all protein coding genes in humans [6, 7]. A majority of miRNAs are evolutionally conserved in closely related species [8] suggesting that they might have conserved functions. Predictions based on computational approaches indicate that each miRNA can target hundreds of genes simultaneously, while an individual gene transcript can be subject to regulation by many miRNAs [9, 10].

The miRNAs are critically involved in many aspects of retinal development, homeostasis, and pathobiology. In this review, we will describe recent advances in knowledge of the role of miRNAs in retinal health and diseases with a primary focus on common retinal pathologies. In addition, we will discuss the postulated roles of circulating miRNAs and their prospective values as biomarkers of retinal degenerative disorders.

## 2. miRNA Signatures and Retinal Disorders

In recent years, transcriptome analyses have affirmed the existence of considerable abundance of miRNAs expressed in the retina [11–15]. This relative abundance is likely due to the tight regulation required for adjustment of the sensory organ to a wide dynamic range of visual stimuli and a demanding physiological environment. Studies suggest that miRNAs play essential roles in retinal development, survival, and normal function. Thus, the absence of Dicer, a principal enzyme in miRNA biogenesis, leads to reduced expression of miRNAs in the retina. Deletion of Dicer alters the function and survival of retinal neurons and leads to severely impaired vision [16–18]. Dicer conditional deletion obstructs the progression of retinal progenitor cells to late cell types, leading therefore to the indefinite formation of early cell types [19]. A recent study elucidated the molecular mechanism of this phenomenon, indicating that three miRNAs (let-7, miR-125, and miR-9) are controlling the transition from early to late stage retinal progenitors [20].

We now know that there are more than 250 miRNAs that are differentially expressed in developing and mature mouse retina [11, 14] some of which are present ubiquitously and others are expressed in a cell specific manner [12, 14]. The abnormal expression or activity of numerous retinal miRNAs has been linked to the etiology of common retinal disorders such as age-related macular degeneration (AMD) [21, 22], diabetic retinopathy (DR) [23–27], retinitis pigmentosa [28–30], and retinoblastoma [31–34] in both human and animal models (see Table 1).

Transgenic mice, with the miR-183/96/182 cluster inactivated, displayed gradual synaptic connectivity defects of the photoreceptors resulting in their extreme sensitivity to light and severe retinal degeneration [35, 36]. These results indicate a protective role of the miR-183/96/182 cluster in retinal neurons. In Xenopus, miR-124 controls the proper path finding of retinal cone photoreceptor cells [37] whereas miR-124a has been found to be essential for maturation of developing cone cells in mouse retina [38]. In oxygen-induced retinopathy (OIR) mice model, the intravitreal injection of miRNA-126 deterred the neovascularization process caused by retinal ischemia [27]. In the brains of miR-132/212 knockout mice, the roles of these miRNAs were associated with the control of function and plasticity of synapses [39]. Two miRNAs (miRNA-200c and -150) played an important role in endothelial cell differentiation and chick embryonic blood vessel formation [40]. Also in the brain, miRNAs have been implemented in vasculogenesis and strength of the vasculature. For example, Mir-211 was found to regulate the expression of Angiopoietin-1, which is involved in stabilization of the vasculature and its resistance to pathological factors such as inflammation and leakage [41]. In the mouse retina, mir-218 represses the expression of Robo1, Robo2, and glucuronyl C5-epimerase (GLCE), required for normal vascularization of the retina and therefore influences vascular guidance of blood vessels during retinal development [42]. Angiogenesis repression in endothelial cells seems to be controlled by miR-24, which simultaneously regulates multiple components in the actin cytoskeleton pathways [22].

Glaucoma is a multifactorial disease and its cure constitutes a great challenge. Retinal ganglion cell (RGC) death is critical element in the pathophysiology of all forms of glaucoma leading to the irreversible loss of functional capacity of the RGCs and, finally, loss of vision [43]. Since replacement or substitution of RGCs is not yet possible, neuroprotection is a main component of every treatment. It has been proposed that miRNAs might play a role in the regulation of neuronal cell death (reviewed in [44]) and in regulation of apoptosis during normal development in the retina [43]. Thus, the possibility exists that miRNA-mediated neuronal death may also play an important role particularly in devastating retinal disorders.

Taken together, these reports indicate that miRNAs are of importance to the etiology of various retinal pathologies and thus their balanced expression may be important for fine-tuning retinal health. Changes in miRNAs expression may be affiliated with onset and progression of disease and therefore miRNAs profiling might have relevance to early detection of retinal injury and prediction of progression of chronic retinal diseases.

### 2.1. Cell-Specific miRNA Signatures in Retina

It is a great challenge to approach the question whether every cell type in the eye has its own miRNA phenotype (miRNome), since one miRNA can target a plethora of genes that might be expressed in different retinal neurons. However, miRNAs are reported to be differentially expressed in neural retina and therefore their expression patterns could possibly be used as signatures to distinguish retinal cell types (Table 2). For example, miRNA 204 is strongly expressed in RPE, while miRNA 124 has been found in all cell layers of the neuroretina but is not detected in the RPE [45]. Further, miR-133b is specifically expressed in retinal dopaminergic amacrine cells [46], whereas miR-29b is found in RGCs and cells of the INL [47]. Two miRNAs (miR-204 and miR-211) are exclusively expressed, in vitro, in the RPE and contribute to its functional maturation [48]. The miR-183/96/182 cluster has been found in photoreceptors and interneurons of the inner nuclear layer [35]. We might predict then that the presence or absence of particular miRNAs might serve as molecular markers for characterizing the normal/abnormal functions of particular retinal cells within the retinal tissue. For example, Lin and colleagues suggested that miR-23a expression might be necessary for the maintenance of a healthy RPE because downregulation of miR-23a was observed in AMD, whereas its upregulation protects RPE cells from oxidative damage in ARPE19 cells [49].

Microglia cells are essential for the maintenance of the CNS, where they clear debris, derived, for example, from neuronal apoptosis and they can interact with and modify the structure of synapses [50]. The prevailing phenotype of microglia in neuronal tissues is known as a resting phenotype. However, following an injury such as ischemic insult, microglial cells become activated and give rise to different subset of cells with distinct morphology and function [51]. Among the miRNAs, miR-155 and miR-124 have been associated with switching from resting microglia to the activated M1 or M2 macrophage phenotypes in the brain (reviewed in [52]). It is thought that the M1 state is associated with a proinflammatory pathway leading to cytotoxicity and tissue injury, whereas the M2 state is associated with immune suppression, angiogenesis and tissue repair [53, 54]. Resting microglia cells express low levels of miR-155 and relatively high levels of miR-124. In contrast, activation state M1 is observed with low expression levels of miR-124 and relatively high levels of miR-155. Conversely, changes in abundance of these miRNAs (miR-124 upregulated and miR-155 downregulated) are associated with the activation state, M2. Therefore, manipulation of miRs like miRNA-124 and miRNA-155 could be used as new therapeutic tools for regulating specific gene targets within microglia and hence microglial cell behavior in the CNS. In the retina, the microglia reside in the layers between the nuclear layers [55] and the state of microglial activation would provide a good indication for ongoing retinal injury-related inflammation or recovery from injury. Future studies of retinal disorders may usefully assess and quantify miRNA-124 and miRNA -155 as modulators of the M1 and M2 status of the retinal microglia.

### 2.2. Circulating miRNAs as Biomarkers for Retinal Disorders

It is particularly noteworthy that miRNA transcripts have been identified in almost all body fluids: plasma [56, 57], serum [32], cerebrospinal fluid [58], urine [59] and saliva [60]. Within the eye miRNAs have been detected in the vitreous [61] and in the aqueous humor [62]. Weber and colleagues analyzed 12 different body fluids and identified 600 miRNA species [63]. Their concentrations in the fluids are significant, although the relative abundance of the individual miRNAs varies widely among the different fluids. While 61 miRNAs were common among all fluids, fluid-specific miRNAs were also detected. For example, miR-637 is specific for tears, miR-193b for breast milk, miR-224 for plasma, and miR-508 for seminal fluid. Interestingly, a large number of miRNAs were detected in tears and the authors postulate an important role of those miRNAs in maintaining and/or regulating the normal function of the eye.

At present the knowledge about the origin or functions of circulating miRNAs is very limited. It has been shown that altered miRNA expression patterns in the fluids might be the cause or the result of various disease states. Several recent studies are beginning to demonstrate the possibility of making use of circulating miRNAs as biomarkers for detection of biological conditions or severity of disease progression. Recently, blood-derived miRNAs have been established as biomarkers for ischemic heart diseases [64], multiple sclerosis [57], human cancer [40, 59], diabetes [65], and liver injury [66].

How miRNAs end up in the body fluids is not entirely clear. Studies have suggested passive leakage into the circulating blood stream [67] as well as active cellular export [68]. Interestingly, the miRNA transcripts are more resistant to degradation than mRNA transcripts when exposed to room temperature and freeze-thaw cycles [69]. In addition, they seem protected from RNAse and possess remarkable stability in the blood stream. There is evidence that miRNAs are loaded into different type of microvesicles to be transferred between cells and these physiological carriers protect them from degradation [70–72]. Furthermore, the expression profiles of exosomal miRNAs have been suggested recently as biomarker for diagnosis of bipolar disorder and schizophrenia [73]. Also microvesicles originating from endothelial progenitor cells are reported to protect the kidney from ischemia-reperfusion injury by microRNA-dependent mechanism [74]. Circulating miRNAs in microvesicles and/or exosomes have a great potential for the diagnostics and prognostics of human diseases [75]. Other studies described nonvesicle associated miRNAs transported by protein carriers, such as Argonaute-2 [76], nucleophosmin-1 [77], or high-density lipoproteins [68]. It is possible that there are other proteins that form complexes with miRNAs to ensure their protection during their transit in the body fluids.

Despite remarkable findings in other systems, there are no studies at this time reporting on the presence of circulating miRNAs associated with retinal disorders. However, Ragusa and colleagues analyzed the vitreous humor (VH) from patients affected by various ocular diseases [61]. Using TaqMan Low Density Array (TLDAs), the authors profiled the transcriptome of 745 miRNAs in vitreal and serum samples. They identified 92 out of 745 circulating miRNAs in the VH and some miRNAs had expression levels that were more than 100-fold different when compared to serum. More importantly, some vitreal miRNAs were differentially expressed in patients with different ocular diseases and in comparison to the values detected in the healthy donor serum. There were miRNAs abundantly expressed in VH but not detected in serum. This finding indicates that some miRNAs are specifically secreted in the eye and it might be hypothesized that the vitreous miRNAs may be part of an intercellular communication system within the tissues of the eye. In another recent study, Dunmire and coworkers analyzed the aqueous humor (AH) from human subjects undergoing cataract surgery [62]. The authors used miRNA PCR array platform that represents 264 human miRNA sequences and detected 110 miRNAs in the aqueous humor. Since there are 1527 miRNAs identified in the human genome so far and the authors only assayed for 264, they proposed that the total number of miRNAs in the AH might be much higher. Unlike blood, the internal eye fluids, such as the aqueous and vitreous humors, might contain higher concentration of secreted retinal miRNAs, but analyses of these fluids would involve invasive procedures. However, the finding that a good portion of the vitreous miRNAs are also present in plasma and are differentially expressed in patients with different ocular disorders is compelling evidence that miRNAs associated with retinal disorders could also be detected in the blood stream and their fingerprints could be associated with retinal damage. Such blood-borne miRNAs may be excellent and less invasive biomarkers for monitoring retinal health. In support of this speculation, some studies provide evidence that some of the miRNAs that are upregulated in ischemic brain can be detected in the blood [78].

It would be interesting to explore if some of the miRNAs that significantly changed their expression in response to ischemic insult can be detected in plasma in early stages after the insult. This would suggest that circulating miRNAs are suitable for noninvasive indicators and much needed biomarkers, for early detection of retinal disorders. This is essential because prompt verification might be able to advance clinical management and improve long-term outcomes in visual health.

### 2.3. miRNA Based Therapeutic Approaches in Retinal Diseases

The use of miRNA-based approaches for treatment of retinal pathologies has potential due to the relative accessibility of the retina for gene delivery. Gene therapeutic techniques have already been implemented in treatment of retinal disorders in human [80, 81]. Also, in mouse models of recessive retinal degeneration, recombinant adeno-associated virus (rAAV) vectors were successfully used for gene delivery [82]. Furthermore, rAAV-mediated approach was used, for precise subcellular targeting of retinal neurons such as RGCs in vivo [83]. Recent advancements have been reported in nonviral gene therapy in targeting diseases of cornea, retina, and retinal pigment epithelium (reviewed in [84]). In one study, Karali and colleagues combined the advances in the rAAV approach for gene delivery, with the knowledge of cell-specific miRNA signatures in retina, and developed a novel approach to restrict transgene expression in a particular retinal cell type [45]. Using a tandem of recognition sequences for miRNA-204 (strongly expressed in the RPE) and miR-124 (expressed in all retinal cell layers, but not detected in the RPE), the authors selectively prevented the AAV2/5-mediated expression of exogenous GFP in the RPE and photoreceptors, respectively. The approach was validated in the retinas of mice and pigs and might be used to either restrict a robust expression mediated by ubiquitous promoter or to provide additional expression control when using cell-specific promoter.

The miRNAs have great potential in gene therapeutics, since they have the ability to target multiple genes simultaneously and, furthermore, these genes might belong to a single signaling pathway. Therefore, manipulation of miRNAs can affect many components of a pathway as opposed to the gene-specific manipulation achieved by gene delivery or replacement. On the other hand, several coexpressed miRNAs can collectively control a given biological process by regulating shared key molecules of that process. Clear examples of this type of multilevel regulations in retinal disorders are yet to come. However, specific miRNA activity can be inhibited by several available methods, which involve delivery of synthetic miRNA mimics and antagonists (antagomirs). Such strategies have been used to manipulate miRNAs expression in inner retina and to determine if their upregulation and downregulation establish them as reliable drug targets. For example, in a mouse model of oxygen-induced retinopathy treated with an intravitreal injection of miRNA-126 (plasmid pCMV-MIR-126/liposome mixture), there is inhibition of retinal neovascularization induced by ischemia [27]. Also, it has been shown that intravitreal injection of the miR-200b-mimic prevented diabetes-induced increase in VEGF production, while miR-200b antagonist led to increased VEGF accumulation in rat's retina [24]. Furthermore, Silva and coworkers report that overexpression of miR-29b in STZ-induced diabetic rats may be protective against apoptosis of RGCs and neurons of the INL via the proapoptotic RNA-dependent protein kinase (PKR) signaling pathway [47]. These authors show that miR-29b is indirectly targeting RAX (PKR-associated protein X), the only known physiologic activator of PKR, and could activate apoptosis by activating the PKR-signaling pathway. Based on the finding that RAX is negatively regulated by miR-29b and may represent a mechanism of protection of retinal neurons against apoptosis, it would be reasonable to propose that intravitreal delivery of miR-29b mimics might be another valuable approach for treatment of diabetic retinopathy.

MiRNAs may be involved in transferring inflammatory signals between cells and may have prognostic value in diseases in which there are common inflammatory mechanisms. For example, Li and colleagues reported secretion of possibly pathogenic amounts of two miRNAs (miRNA-146a and miRNA-155) from stressed human primary neural cells causing downregulation of an important repressor of the innate immune response, complement factor H (CFH) [21]. Adding these two miRNAs to conditioned medium induced CFH deficit in brain cells and these effects were reversed using anti-miRNA approaches. Both microRNAs are found in the neocortical extracellular fluid (ECF) and in the cerebrospinal fluid (CSF) of Alzheimer's disease patients. Remarkably, CFH-mediated inflammatory degeneration is characteristic of both Alzheimer's disease (AD) and age-related macular degeneration (AMD) and miRNA-146a and miRNA-155 are upregulated in both diseases [21]. These secreted miRNAs might be involved in cell to cell signaling and contribute to the propagation of AMD and AD over time.

## 3. Regulatory Networks of miRNAs and Transcription Factors

**Figure 1:**
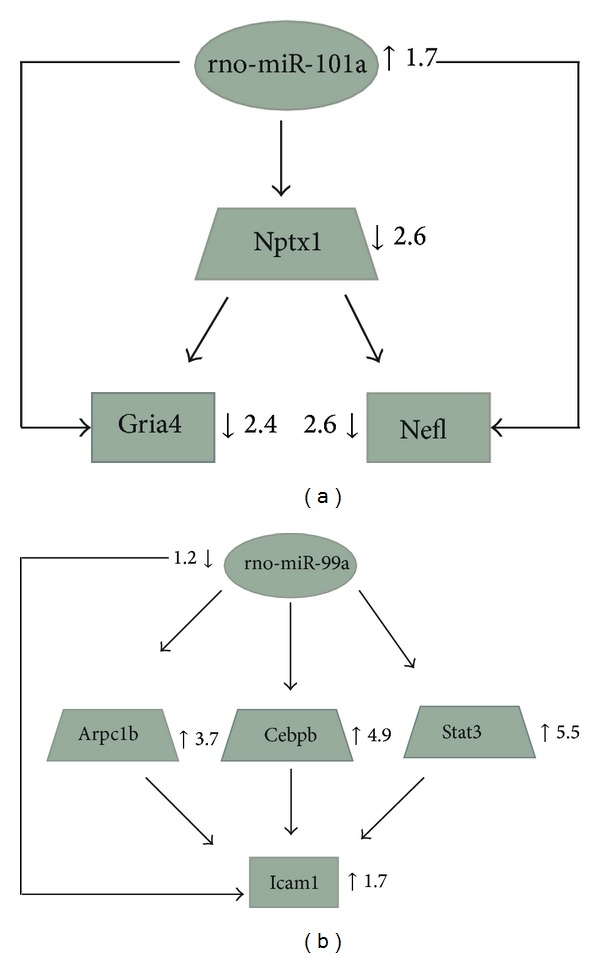
Two examples of mixed regulatory circuits, each containing a miRNA (oblate spheroid), linked to TFs (trapezoids) and coding gene targets (rectangles), all of which changed their expression in response to transient ischemia-reperfusion injury in the retina. The numbers and bold arrows represent fold change relative to sham control animals and up- /downregulation (resp.) of the miRNAs, TFs, and protein coding genes implicated in the mixed regulatory circuits. (a) Direct and indirect downregulation of two coding genes by the same miRNA (rno-miR-101a). At 24 h IR period, rno-miR-101a targets the protein coding genes Gria4 (glutamate receptor) and Nefl (neurofilament) directly. At the same time, rno-miR-101a can control these genes indirectly by targeting the TF Nptx1, which in turn targets Gria4 and Nefl. (b) miRNA targets several TFs which act together to coordinate the reduced expression of a single gene. At 24 h IR period, rno-miR-99a targets the protein coding gene Icam1 (adhesion molecule) directly. At the same time, rno-miR-99a can control this gene indirectly by targeting 3TFs (Arpc1b, Cebpb, and Stat3), which in turn target Icam1.

Transcription factors (TFs) recognize and bind to transcription factor binding sites (TFBS) located upstream of miR genes and regulate their transcription in a polymerase II dependent manner [85, 86]. Studies suggest that microRNAs target predominantly TFs rather than other types of protein coding genes and suggest the possible interconnection of these two regulators in regulatory networks [87, 88]. Until recently, little was known about the involvement of miRNAs in regulatory networks but it now seems likely that there is interplay between miRNAs and TFs in gene regulation. Recent studies exploring the links between these major regulators indicate their roles in mixed feedforward and feedback loops, which can be associated with different cellular processes and diseases [89–94]. Current studies have begun to integrate these regulatory loops into subnetworks [95, 96]. Our lab has generated mRNA and miRNA microarray expression data to investigate time-dependent changes in gene expression, following induction of ischemia-reperfusion (IR) injury in the rat retina. Analyses of the data show that many of the TFs that change their expression in response to IR injury had common targets with miRNAs, whose expression was also altered by the IR injury paradigm. For example, at the 24 h reperfusion period following ischemia, rno-miR-101a targets the TF, Nptx1, and both of these regulators cotarget the protein coding genes Gria4 (glutamate receptors) and Nefl (neurofilaments) (Figure 1(a)). It is possible that induction of this interactive loop by up-regulation of the microRNA down-regulates the TF as well as the two target genes. Studies have already implicated several of the components of this loop in eye disorders. For example, Nptx1 is a member of the neuronal pentraxin (NP) gene family, which has a role in synaptic development. A knockout mouse study in which the NP is eliminated indicates that neuronal pentraxins play a role in *α*-amino-3-hydroxy-5-methyl-4-isoxazolepropionic acid receptor (AMPAR) mediated transmission in vivo, raising the possibility of involvement of NP in synaptogenesis in normal development of the visual system [97]. The neurofilament genes (NEFH, NEF3, or NEFL) have been previously shown to be markers of ganglion cells [98] and have been implicated in the maintenance of myelinated peripheral nerve fibers [99].

We have found evidence of other types of regulatory loops from our IR-injury associated datasets. For example, a single miRNA, miR99a, targeted 3 TFs (Arpc1b, Cebpb, and Stat3) and together they regulate the Icam1 gene (adhesion molecule) (Figure 1(b)). They all cooperatively regulate the Icam1 gene (adhesion molecule) (Figure 1(b)). In this scenario, downregulation of the miRNA is the most likely reason for the upregulation of the targeted TFs and the Icam1 gene.

In the analyses of our IR associated datasets, we define the regulatory loops as closed loops if they contain a TF and a miRNA in which both target one or more common genes and in the same time the miRNA targets one or more TFs. These closed loops serve as important motifs in gene regulatory networks. In our preliminary results, we have identified 87 closed loops at the 24 h post-IR time point and 140 closed 13 loops at the 7 d post-IR time point.

**Figure 2:**
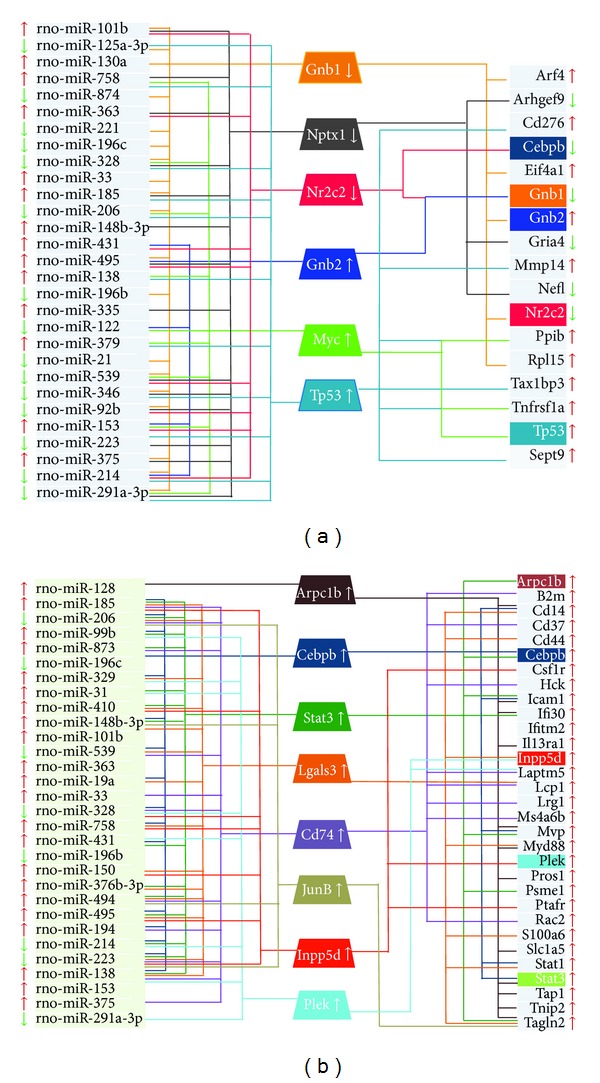
Regulatory subnetworks for two time points following transient retinal ischemia-reperfusion injury. The networks contain three types of molecules: miRNAs (shown in the left column), TFs (shown as trapezoids in the center), and coding gene targets (shown in the right column). All of these molecules changed their expression in response to IR injury twofold or more, relative to their sham controls. The arrows next to each gene correspond to gene regulation: uppointed arrows indicate increased gene expression; downpointed arrows indicate decreased gene expression. TFs, miRNAs, and coding genes that are linked with the same colored line indicate that the miRNAs target the TFs and at the same time, both of these regulators cotarget the coding genes. With the notable exception of Cebpb, there were no common target coding genes or TFs in the 2 subnetworks, even though they shared 19 common miRNAs. (a) Regulatory subnetwork consisting of miRNAs, TFs, and target genes, which changed their expression at the 24 h IR injury time point relative to their sham controls (FC ≥ 2). At this post-IR time point, six TFs were fine-tuned by 29 miRNAs to jointly regulate 17 target genes. (b) Regulatory subnetwork consisting of miRNAs, TFs, and target genes, which changed their expression at 7 days after IR injury relative to their sham controls (FC ≥ 2). At this post-IR time point, eight TFs were regulated by 30 miRNAs to jointly control the expression of 31 target genes.

These closed loops were further integrated into larger regulatory subnetworks. Two sub-networks, corresponding to 24 h and 7 d time points after retinal IR-injury, were generated and are shown below (Figure 2). At the 24 h IR-injury time point, 6 TFs are fine-tuned by 29 miRNAs to jointly regulate the expression 17 target genes. At the 7-day IR-injury time point, 8 TFs were regulated by 30 miRNAs to control the expression of 31 genes. With the exception of Cebpb (CCAAT/enhancer binding protein (C/EBP), beta), there were no common TFs or target genes between the 2 subnetworks. Cebpb is an important transcriptional activator in the regulation of genes involved in immune and inflammatory responses. Although it was common for both post-IR-associated networks, Cebpb had different pattern in each of the time points: its expression level was decreased at 24 h and increased at 7 d after IR-injury. Although the number of miRNAs involved in the subnetworks at 24 h and 7 d is very similar (29 versus 30 miRNAs, resp.) there were 19 common miRNAs between both networks and they were found to regulate different sets of TFs and target genes. Recent studies have suggested that tissue specific miRNAs tend to regulate different sets of targets, whereas trivial miRNAs are more likely to coregulate the same set of targets [100]. However some of the 19 miRNAs seen in our study have previously been associated with endothelial cells, cardiac tissue, breast cancer, liver cancer, and lung cancer [101–105].

According to our preliminary results, it is plausible to propose that different circuits might be executing different functions at different time periods, perhaps in different cell types, after IR-injury. The purpose of our ongoing study is to identify specific “signature-modules,” the manipulation of which, might influence the etiology of ischemic-related diseases. and progression of retinal ischemic-related diseases. Further analyses to experimentally validate some of these regulatory modules and identify the biological significance of their coordinated regulation in healthy and diseased retina are in progress.

The future characterization of regulatory networks in various experimental contexts will be essential for better understanding of how regulators coordinately contribute to the diverse dynamic processes occurring in retina in the normal and pathological states.

### 3.1. Control of miRNA Abundance, Stability, and Degradation

Understanding the cause and consequence of the dynamic changes in mature miRNA levels in diseased and healthy retina requires in-depth knowledge not only of their synthesis but also of how specific miRNAs are destabilized and targeted for elimination.

In vivo and in vitro studies have demonstrated that internal loops structures of primary transcripts (pri-miRNA) drive bidirectional processing, which affects the abundance of mature miRNAs in both plants and animals [106, 107]. Thus the balance between the productive and abortive processing of miRNA precursors is a possible mechanism for dynamic regulation of miRNA levels. De et al. It has been shown that binding of miRNAs to Argonaute (“loading”) has a protective effect, while dissociation of miRNAs from Argonaute (“unloading”) is promoted by highly complementary target mRNAs [108]. The authors of this study propose that all miRNA degradation pathways have to contend with this high-affinity interaction between Argonaute and its bound miRNA.

Recent studies have linked the control of miRNAs stability and degradation to two proteins, the atypical poly(A) polymerase PAPD4 and the 5′-to-3′ exonuclease XRN2 [109, 110]. These proteins have antagonistic mode of action on miRNAs: PAPD protein regulates the length of the poly(A) tail of miR transcripts and influences their stability, while XRN2 is involved in degradation of unprotected mature miRNA. PAPD and XRN2 proteins have been shown to be present in hippocampal neurons where they may be associated with cognitive functions, such as memory and learning [111]. Furthermore, this latter study suggests that the balance between stability and degradation of miRNAs is a new mechanism for posttranscriptional miRNAs control in the nervous system.

In the context of the retina, PAPD and XRN2 were found to be expressed mainly after neuronal differentiation and do not accumulate in retinal progenitor cells [112]. They have been identified in wide variety of neurons such as horizontal, amacrine, and ganglion cells, while low expression levels were observed in microglia, endothelial cells, and astrocytes [112]. The functional regulation of these genes appears to be posttranscriptional, since upregulation of PAPD protein was obtained after few hours of dark adaptation, while no changes in gene expression level were detected. Since dark adaptation regulates PAPD but not XRN2, the authors of this study suggest that the ambient light causes changes in the balance between stability and degradation of miRNAs, which is a mechanism for control of miRNA expression in retinal neurons.

It would be interesting to investigate how many retinal miRNAs change their expression levels in response to light and if they are involved in fine-tuning the stability-related genes.

## 4. Current Challenges and Future Directions

Despite the recent advances in the field, there is still a need for additional research before the full biological relevance and clinical potential of miRNAs can be realized. Future studies need to provide supporting evidence for the choice of either single or multiple miRNAs whose effect(s) would be sufficiently powerful to treat a particular retinal disorder.

### 4.1. Technical Challenges

There are numerous unresolved issues regarding the identification, functional role, and contribution of miRNAs to retinal diseases and/or disorders. For example, reliable standard methods for miRNA isolation and accurate quantitation are crucial requirement. So far the quantitative characterization of miRNAs relies on RT-PCR and depends on the presence of endogenous unchanging miRNAs and on the amplification efficiency of primers. Discrimination of single nucleotide differences by PCR is not always possible. In addition, PCR-based methods quantify miRNA precursors as well as the mature active miRNA. Although the proportion of pri-miRNA versus mature miRNA in the cell has not yet been extensively evaluated, there are studies suggesting poor correlation between expression of miRNA primary transcripts and mature miRNA [113]. Possible posttranscriptional modifications of miRNAs, which might influence their activities, cannot be captured by PCR techniques.

Microarray-based analyses allow parallel screening of thousands of miRNAs in one sample and are a powerful tool for selection of differentially expressed miRNAs. However, the concentration of putative miRNA biomarkers in the bloodstream is very low and microarray technology might not be suitable for detection of miRNAs in the circulation. Next-generation sequencing (NGS) based approaches might provide an alternative strategy for identification of more miRNAs associated with variety of chronic retinal diseases. NGS technology does not rely on predesigned primers or probes. Thus Djebali and colleagues used ultra-deep sequencing of RNAs in 15 different cell lines, and they reported that 75% of the cellular genome is transcribed at different periods during the cell's life cycle [114]. This and other studies based on the use of NGS and various prediction algorithms discovered numerous new miRNAs and the authors predicted that many more remained to be discovered before the whole human miRNA transcriptome is considered complete [115].

A major challenge toward using miRNAs as therapeutic agents would be the avoidance of toxicity derived from off-target effects. Therefore, it is clear that the prediction algorithms for identification of miRNA target genes need further development to decrease the rates of false positive targets. The low consensus between major prediction algorithms, such as PicTar, miRanda, and TargetScan, indicates the need of some standardization for the prediction of miRNA targets [116].

### 4.2. Targeted Regulation of miRNA

**Figure 3:**
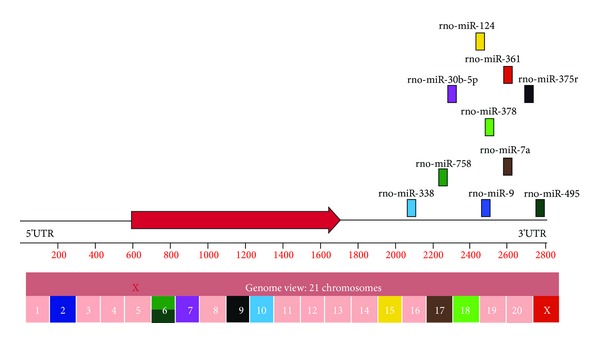
The 3'UTR of the intronless Forkhead Box E1 gene (*foxe*1), located on chromosome 5 (**X**), is targeted by 10 miRNAs (rectangles with different colors). All the miRNAs are localized on different chromosomes in the rat genome (colored chromosome corresponds to miRNAs of the same color). The 10 miRNAs are upregulated in response to retinal IR-injury in rat retinal ischemic model while the expression level of *foxe*1 is reduced.

The transient transfection of miRNA mimics or inhibitors has been successfully implemented in mouse retina [20] and human RPE cells [30] and is attractive strategies to treat retinal degenerative diseases in humans. Most examples from the literature manipulate the expression of a single retinal miRNA. However, silencing a single miRNA might not be sufficient in certain retinal disorders due to their multifactorial nature. Also genes can be targeted by multiple miRNAs (as shown in Figure 3) and modulation of gene expression might require manipulating combination of miRNAs. Scientists in this field have implemented multi-target anti-miRNA antisense oligonucleotides (MTg-AMOs), which consist of multiple antisense sequences combined into a single unit to target several microRNAs simultaneously [117]. MTg-AMOs have been successfully used in cancer research for inhibiting expression of oncomiRs [118]. Future studies could design MTg-AMOs to inhibit activities of miRNAs that control key aspects in common retinal pathologies, for instance, induction of apoptosis. Another innovative strategy for regulation of miRNAs activities would be the use of the naturally occurring circular RNA macromolecules. Recent studies have shown that these molecules can serve as miRNA sponge and led to ultimate miRNA deactivation [119, 120]. Vectors expressing these circular molecules might be a new way to inhibit miRNAs activities in retina and have therapeutic benefits.

### 4.3. Circulating Biomarkers and Their Putative Functions

Circulating miRNAs might be passive by-products of cell death having no biological function, or they could be messengers, activating as yet undiscovered systemic responses. In both of these cases, they could be reliable biomarkers for retinal pathologies in a clinical context. However the mechanisms by which miRNAs are loaded and transported in vesicles or become associated with carrier proteins are not fully elucidated. It is largely unknown how particular miRNAs are “labeled” for export. Additional studies are needed to clarify the ratio between vesicle- and protein-associated circulating miRNAs, for instance, diseased and healthy retina, to provide a basis for more accurate miRNAs extraction and more responsive biomarker assays. A recent report suggested that the mechanism of delivery of miRNAs in the blood might depend on the kind of injury. Using distinct mouse models for liver diseases, where the liver damage is caused by alcohol, drugs, or inflammation, miRNA-122 and miRNA -155 were abundant in the exosome-enriched fraction when the liver damage is caused by alcohol or inflammation, while in drug-induced liver injury model both miRNAs were detected mainly in the protein rich soluble fraction [121]. The authors suggested that differentially packaged miRNAs originate from different cell types and have different functions and targets.

In addition to the issue of packaging, another area for future exploration is the use of miRNAs as possible messengers for intercellular communications within the retina. If miRNAs can be transferred and utilized by receiver cells, they might offer an elegant way to coordinate the intracellular activities of those cells. For example, Hergenreider and colleagues demonstrated in an in vitro study, that extracellular vesicles shuttle miRNAs from endothelial cells to smooth muscle cells [122]. Furthermore, miRNAs transferred by exosomes are functional in the receiver cell [123] and are capable of triggering downstream signaling events. Taken together, elucidating the mechanisms of entry in the circulation, packaging, delivery, and recognition of endogenous miRNAs would open opportunities for developing miRNA-based approaches for cell to cell communications. This area of research is largely unexplored and yet it could greatly expand our understanding of the impact of miRNAs based cell communications within the retina.

In conclusion, the miRNA represents an exciting area of focus for future discoveries in retinal research. The association of specific miRNAs with particular diseases/disorders of the retina remains a largely unexplored area. Retinal miRNAs in the peripheral circulation may serve as markers of disease stage or monitoring therapeutic response or disease recurrence. In addition to their use as biomarkers, there is a great potential to manipulate miRNAs in the retina to implement cellular health and ultimately improve visual function.

## Figures and Tables

**Table 1:** Differentially expressed microRNAs in retina related disorders in human and animal models.

MicroRNA	Organism	Retinal disorder	Refs
miR-9, miR-125b, miR-146a, miR-155 miR-24	Human Endothelial cells (EC)	Age-related macular degeneration	[21, 22] [22]
miR-126, miR-146, miR-200b, miR-146a, miR-182, miR-96, miR-183, miR-211, miR-204, and miR-124, miR-10b, miR-10a, miR-219-2-3p, miR-144, miR-338 and miR-199a-3p	Rat	Diabetic retinopathy	[23–27]
miR-365b-3p, miR-17, miR-18a, and miR-20, let-7 miR-376a	Human Retinoblastoma cells	Retinoblastoma	[31–33] [34]
miR-1, miR-133, miR-96, miR-183, miR-376, miR-691 miR-146a, miR-146b-5p	Mouse Human RPE cells	Retinitis pigmentosa	[28, 29] [30]
miRNA-183/96/182 cluster	Mouse	Retinal degeneration	[35]
miRNA-34a	RPE cells	Inhibits proliferation and migration of RPE cells	[79]
miR-150 and miR-200c	Human embryonic stem (hES) cell Chick embryos	Endothelial cell differentiation Blood vessel formation	[40]
miR-126	Mouse	Reduce ischemia-induced retinal NV	[27]

**Table 2:** Differentially expressed microRNAs in retinal cell types.

MicroRNA	Regulation/function	Retinal cell type	Refs
miR-204, miR-211	Upregulated	RPE	[48]
miR-23a	Upregulated (in healthy cells) Downregulated (in AMD)	RPE	[49]
miR-183/96/182	Downregulated in dark Upregulated in light	Photoreceptors and interneurons of the inner nuclear layer	[36]
miR-29b	Upregulated after streptozotocin (STZ) injection; upregulation protects against apoptosis	RGCs and cells of the INL	[47]
mir-133b	Overexpression suppresses maturation and function of dopaminergic amacrine cells	Dopaminergic amacrine cells	[46]
mir-124	Abundantly expressed in differentiated neurons	All retinal cell layers, but is not detected in the RPE	[45]
miR-155*, miR-124.	**Resting microglia**: low expression of miR-155 and high expression of miR-124 **Activation phenotype (M1)**: downregulation of miR-124, upregulation of miR-155 **Activation phenotype (M2)**: upregulation of miR-124, downregulation of miR-155	Microglia	[52]

*Expression of these miRNAs has been reported for microglia of the brain.
